# 

**Published:** 1875-11

**Authors:** 


*CONTENTS:*

| | PAGE |
|---|---|
| How to Prevent Colds | 329 |
| Wearing Flannel | 330 |
| The Air We Breathe | 331 |
| School Outrages | 331 |
| Why We Die | 332 |
| Sympathy for the Erring | 332 |
| Our Summer at Maplewood. Chapter X, Odds and Ends | 333 |
| Weak Food | 343 |
| Meat and Poison | 344 |
| Fine Flour Bread | 345 |
| Thanksgiving Day | 347 |
| Worthless Drugs | 348 |
| The Teeth | 349 |
| Curable Diseases | 350 |
| Granulated Wheat Biscuit | 350 |
| Sickness Prevented | 351 |
| Young Men's Christian Association | 352 |
| The Flour of the Family | 352 |
| The Business Outlook | 353 |
| One Woman's Work | 354 |
| Packer's Products | 355 |
| Woman's Temperance Convention | 355 |
| True Temperance | 356 |
| Caution | 356 |
| Poisoning Pickles | 357 |
| To Invalids | 358 |
| Remarkable Gloves | 358 |
| Two Thanksgivings | 359 |
| The Ballot for Women | 360 |
| The American Popular | 360 |
| Renewal of Subscriptions | 360 |
| A Good Woman's Thoughts | 362 |
| A Word for Woman | 363 |
| Still Water | 364 |
| Science in Story | 365 |
| Lectures to Ladies | 365 |
| How to Sweep a Room | 366 |
| Respect the Burden | 367 |
| Our Own | 368 |
| A Great Achievement | 369 |
| Hygiene for the Aged | 372 |

